# Epidemiologic and Clinical Progression of Lobomycosis among Kaiabi Indians, Brazil, 1965–2019

**DOI:** 10.3201/eid2605.190958

**Published:** 2020-05

**Authors:** Marcos C. Florian, Douglas A. Rodrigues, Sofia B.M. de Mendonça, Arnaldo L. Colombo, Jane Tomimori

**Affiliations:** Escola Paulista de Medicina, Universidade Federal de São Paulo, São Paulo, Brazil

**Keywords:** lobomycosis, epidemiology, fungi, mycosis, Lacazia loboi, Indians, Brazil

## Abstract

Lobomycosis is a rare granulomatous skin disease with a high prevalence in the Amazon region. The Kaiabi Indians are an especially affected group. We studied the current epidemiologic and clinical progression of lobomycosis among the Kaiabi in Brazil, from initial case reports in 1965 through 2019. A total of 60 lobomycosis cases had been reported among the Kaiabi, and we identified 3 new cases in our review. Of 550 cases of lobomycosis ever reported worldwide, 11.5% were among the Kaiabi. We note a high incidence among female Kaiabi and a precocious onset of disease in this indigenous population. Male Kaiabi frequently are infected with the multicentric form and women more frequently exhibit the localized form. Ulcerated lesions are observed more often in the multicentric form. The prevalence among this indigenous group could be explained by genetic susceptibility and lifestyle, which exposes them to a particular agent in the habitats in which they live.

Lobomycosis is a chronic and granulomatous fungal disease that affects the skin and subcutaneous tissue. Lobomycosis is classified as a neglected mycosis and is endemic to Latin America, especially the Amazon region (*1*,*2*). It has been reported in travelers who have visited that area but is rarely reported outside this region (*3*,*4*). In 1931, Jorge Lobo published a report of this disease in a nonindigenous man in Recife, Pernambuco state, Brazil (*5*). Since then, the disease has been given many different names, including lobomycosis, Jorge Lobo’s disease, keloidal blastomycosis, and lacaziosis. Of 550 cases reported worldwide, 332 (58.5%) have occurred in Brazil (*6*). 

The etiologic agent of lobomycosis is *Lacazia loboi*, an as-yet uncultured fungus that has phylogenetic and antigenic similarity to *Paracoccidioides brasiliensis*, a dimorphic fungus. *L. loboi* also could be a dimorphic fungus (*1*,*7*). Cutaneous lesions associated with this disease are polymorphic and often clinically appear as a keloid-like nodule. Other manifestations include ulcers, atrophy, tumors, macules, plaques, gummas, scleroderma, infiltrations, or scars; patients can have >1 type of lesion. Lobomycosis can be classified into localized or multicentric forms, depending on the extent of skin lesions. The disease does not usually affect general health, but ulceration or secondary infection can impair quality of life for those affected (*8*). No mucosal or systemic involvement has been reported, but the lymphatic system may be affected, and 1 case involving the testicles has been reported (*9*).

Many dermatologic diseases occur in indigenous people (*10*), but lobomycosis is particularly prevalent among the Kaiabi Indians, an ethnic population that lives in central Brazil. During 1961–1966, most Kaiabi migrated 400 km west from regions where they originally lived (7.3502°S, 58.1383°W) to the Xingu Indigenous Park (XIP; 11.2320°S, 53.1850°W). The migration changed their habits and brought them in contact with other indigenous groups. Most Kaiabi now live in the XIP, an indigenous reserve, providing an opportunity for long-term medical follow-up (*11*).

At different times, 60 cases of lobomycosis have been reported among the Kaiabi (*8*,*11*,*12*). We provide a clinical review and epidemiologic update of all registered cases of lobomycosis among the Kaiabi. In addition, we conducted field visits to the various Kaiabi indigenous villages and identified the current state of lobomycosis in each village, including 3 newly identified cases. We compared the new cases with other reported cases to determine whether other particularities are associated with lobomycosis among this population.

## Materials and Methods

### Study Design and Area

**Figure 1:**
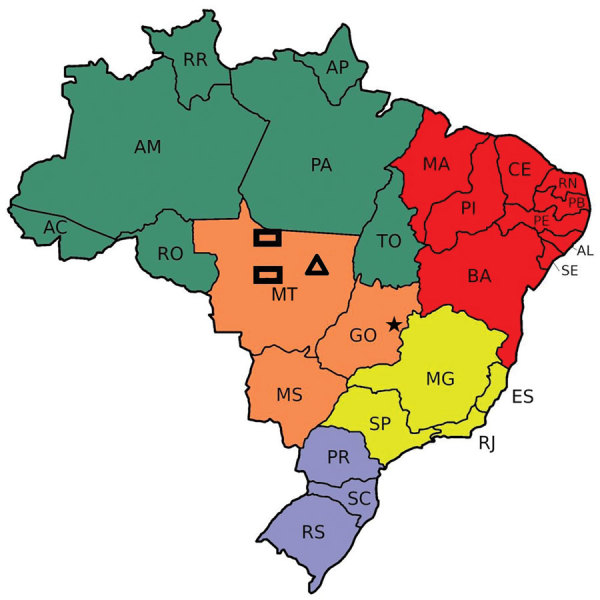
Areas in which the Kaiabi people live in the state of Mato Grosso, central Brazil. Rectangles represent areas near the Arinos and Teles Pires rivers where the Kaiabi originally lived. Triangle represents the Xingu Indigenous Park where many Kaiabi case-patients currently live. Star indicates Brasilia, the capital of Brazil. Green indicates North Region; red indicates Northeast Region; orange indicates West-Central Region; yellow indicates Southeast Region; and purple indicates South Region. AC, Acre; AL, Alagoas; AM, Amazonas; AP, Amapá; BA, Bahia; CE, Ceará; ES, Espírito Santo; GO, Goiás; MA, Maranhão; MG, Minas Gerais; MS, Mato Grosso do Sul; MT, Mato Grosso; PA, Pará; PB, Paraíba; PE, Pernambuco; PI, Piauí; PR, Paraná; RJ, Rio de Janeiro; RN, Rio Grande do Norte; RO, Rondônia; RR, Roraima; RS, Rio Grande do Sui; SC, Santa Catarina; SE, Sergipe; SP, São Paulo; TO, Tocantins.

We conducted a clinical-epidemiologic analysis of reported cases of lobomycosis in members of the Kaiabi ethnic group indexed during 1965–2019. We used a previous extensive review of published lobomycosis cases among the Kaiabi conducted in 1986 (*12*) as the basis of this study (*8*,*11*,*13*). We analyzed medical records obtained during 1965–2019 by the Xingu Project of the Department of Preventive Medicine, Escola Paulista de Medicina, Federal University of São Paulo, São Paulo, Brazil. Each Kaiabi person included in the project had a medical record, and 56 had lobomycosis. We obtained additional unpublished information from the Xingu Project archives and field visits to indigenous villages. We performed an observational follow-up study with information from different time points during 1965–2019. We conducted 2 field visits to reexamine case-patients and identify new cases and analyzed 3 areas in which the Kaiabi have lived (Figure 1). When we did not have access to a medical record, we consulted previous literature reports (*8*,*11*,*12*). 

### Case Definition for Lobomycosis

We used a standard case definition for lobomycosis, which included presence of skin lesions and histopathologic evidence of the disease. Skin lesions related to lobomycosis include keloid-like nodules, plaques, papules, ulcerations, or atrophic lesions. Histopathologic evidence includes the presence of an inflammatory reaction, especially with histiocytic cell infiltrate, and detection of *L. loboi* fungal cells by hematoxylin-eosin and Grocott’s methenamine silver staining.

### Parameters Analyzed

**Figure 2:**
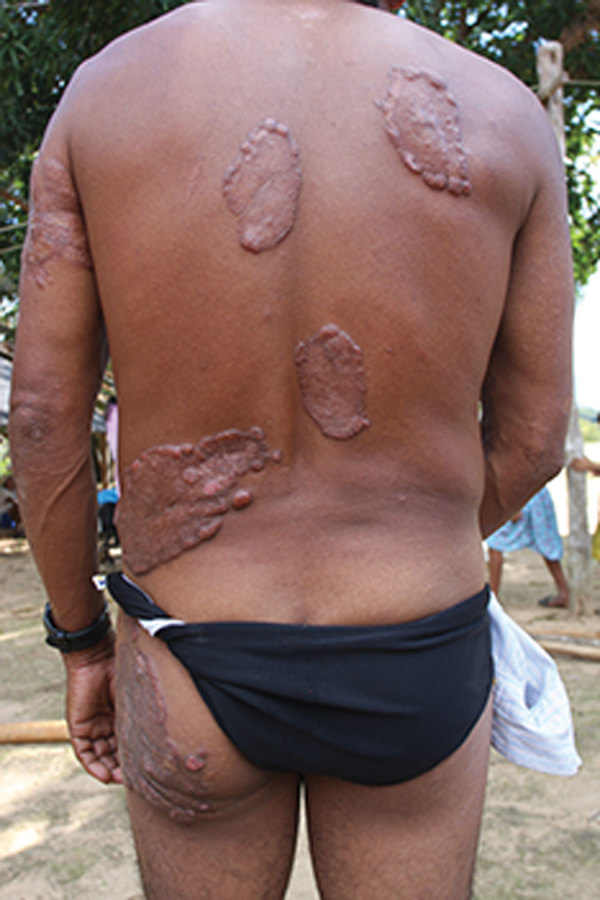
Multicentric lobomycosis affecting the back, buttocks, and arm of a Kaiabi man, Brazil.

**Figure 3:**
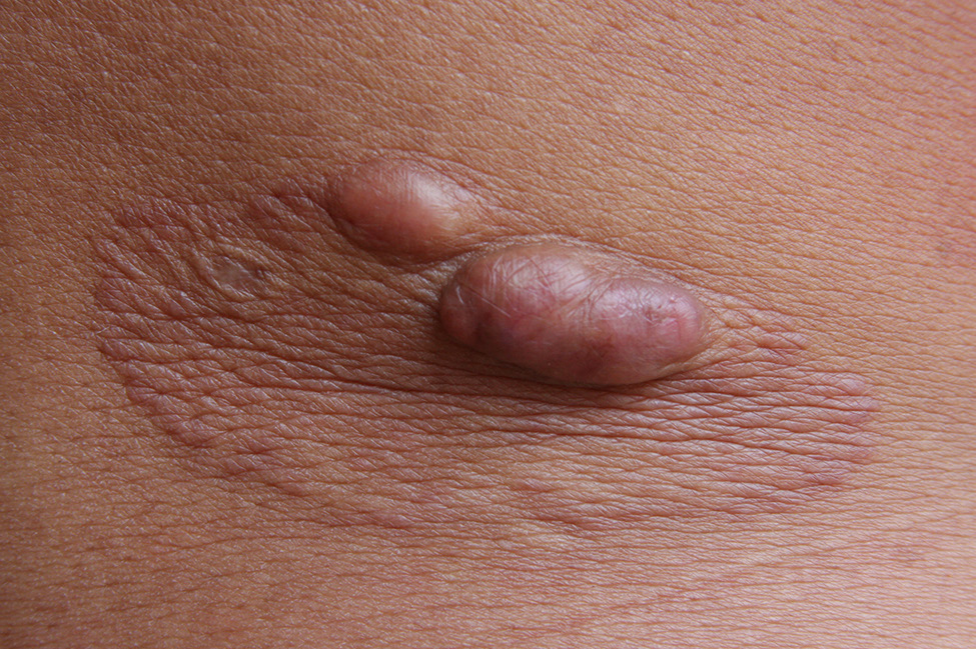
Localized lobomycosis on the arm of a Kaiabi woman, Brazil.

We analyzed the following clinical and epidemiologic parameters: the number of lobomycosis cases among the Kaiabi; the regions in which the Kaiabi currently live and previously lived; sex distribution; age of onset; extent of the lesions; anatomic location of lesions; data concerning the progression of skin lesions; and treatment attempts. We classified cases as multicentric, skin lesions in >1 anatomic area (Figure 2); or localized, lesions restricted to 1 anatomic area (Figure 3).

### Data Analysis

Initial data analysis was descriptive. We also analyzed the prevalence at different times over a 54-year period. For some quantitative samples, we calculated the mean, median, minimum, and maximum values and SDs. We analyzed qualitative variables by calculating absolute and relative frequencies as percentages. We performed inferential analysis by using χ^2^ test for 2 independent qualitative samples, Student *t*-test for independent samples, and analysis of variance for analyzing clinical signs and symptoms and immunologic data. We considered p<0.05 statistically significant.

### Ethics Considerations

This project was approved by Kaiabi indigenous leaders, the Indian National Foundation, and the Brazilian National Research Ethics Committee (registration no. 12776). All participants provided written informed consent before the start of the project.

## Results

### Number of Cases among the Kaiabi 

We identified 63 lobomycosis cases among the Kaiabi from 1965–2019. Of these, 60 were reported previously (*13**–**15*), and we identified 3 new cases. All cases were associated with the case-patients’ original habitat, and none of the reported case-patients were born at the XIP. A temporal series of cross-sectional studies of lobomycosis prevalence reported 51 cases among 400 (12.7%) Kaiabi in 1981. In 2019, a total of 26 cases of lobomycosis were known among 2,242 Kaiabi, a prevalence of 1.16% (Table 1).

**Table 1:** Follow-up observational epidemiologic data on lobomycosis among Kaiabi Indians, Brazil, 1957–2019*

Year	No. reported cases/y	Sex, no.		Total cases	Estimated population	Estimated prevalence of lobomycosis among living Kaiabi population, %
		M	F			
1957	2	2	0	2	ND	NA
1966	12	7	5	14	ND	NA
1967	9	8	1	23	ND	NA
1973	5	4	1	28	180	15.5
1979	22	13	9	50	ND	NA
1981	1	1	0	51	400	12.7
1982	3	2	1	54	500	10.8
1986	2	2	0	56	ND	NA
1989	1	1	0	57	ND	NA
1990–91	2	0	2	59	ND	NA
1994	1	1	0	60	ND	NA
2019	3	0	3	63 (26 alive)	2,242	1.16

*NA, not applicable; ND, no data.

### Regions Where the Kaiabi Live and Have Lived

We obtained information about the regions in north Mato Grosso state in which 41 case-patients originally resided. Twenty-two (53.7%) case-patients were from the Arinos River region, 17 (41.5%) were from the Teles Pires River region, and 2 (4.9%) had lived in both regions.

### Sex Distribution and Age of Onset

Among the 63 case-patients, 39 (61.9%) were male and 24 (38.1%) were female. We obtained information about the age of onset for 38 case-patients. In 24 (63.2%) case-patients, lesions developed before the patient was 21 years of age; in 12 (31.6%), lesions developed at 21–40 years of age; and in 2 (5.2%), the lesions developed at >40 years of age. The earliest age of onset was 1 year of age, and the oldest was 63 years of age. The median age of onset was 18.5 years. We did not obtain data regarding patient age for 25 (39.6%) cases.

### Extent and Location of Lesions

We obtained information on the extent and location of lobomycosis lesions in 62 case-patients, 38 males and 24 females. We noted 34 (54.8%) cases of the multicentric form (Figure 2) and 28 (45.2%) cases of the localized form (Figure 3). The multicentric form more frequently was associated with male sex (Table 2); 27 (71.1%) male patients had the multicentric form and 11 (28.9%) had the localized form. Among female patients, 7 (29.2%) had the multicentric form and 17 (70.8%) had the localized form. For 61 case-patients for whom information on lesion location was available, we noted lesions on the lower limbs in 38 cases, on the upper limbs in 32 cases, on the trunk in 24 cases, on the head and neck in 2 cases, and on the prepuce in 1 case. All patients with the multicentric form had lesions at >1 anatomic site.

**Table 2:** Classification of lobomycosis cases in multicentric and localized forms in relation to sex in Kaiabi Indians, Brazil, 1957–2019*

Clinical form†	Sex, no. (%)		Total, no. (%)
	M	F	
Multicentric	27 (71.1)	7 (29.2)	34 (54.8)
Localized	11 (28.9)	17 (70.8)	28 (45.2)
Total	38 (100)	24 (100)	62 (100)

*Calculations performed by using χ^2^ test (p = 0.001); no information was available for 1 case. †Multicentric, skin lesions in >1 anatomic area; localized, skin lesions restricted to 1 anatomic area.

### Progression of Cutaneous Lesions

During clinical follow-up, 8/18 (44.1%) patients with the multicentric form seen after 20.2 years had new lesions and 2/17 (11.8%) patients with the localized form seen after 28.3 years had new lesions. In addition, 15/19 (78.9%) patients with the multicentric form seen after 12.3 years had lesions with periods of ulcerations and 3/8 (37.5%) patients with the localized form seen after 27.4 years had lesions with periods of ulcerations. We could not perform statistical analysis of these data because of variations in follow-up times.

### Treatment Attempts

Some case-patients received systemic treatment with ketoconazole, itraconazole, or clofazimine, which were not successful. Among 15 cases of localized lobomycosis that received surgical treatment, 6 (40%) cases followed for 30 years had recurrence of lesions. In addition, 2 patients had carcinomatous degeneration in the form of cutaneous squamous cell carcinoma, and both died from metastasis (*8*).

## Discussion

Pereira Filho reported a case of lobomycosis among the Kaiabi in 1957 (*12*,*16*), and Nery-Guimarães reported another in 1964 (*12*,*17*). Since then, many cases in have been reported among this population in the medical literature. The high prevalence of lobomycosis in this population is a notable epidemiologic finding in medical ethnography (*18*). The disease is so prevalent that, since 1915, references have been made to a Kaiabi-associated skin disease, called *piraip* in the Kaiabi language, a branch of the Tupi-Guarani linguistic family. 

The prevalence of lobomycosis in the Kaiabi has been declining over time. Changes in the geographic region in which they live after many Kaiabi moved 400 km east to the XIP, changes in cultural behavior, a lack of fungal agents in the new ecosystem, and changes in the immunity of this population are some hypotheses that could explain the reduction in this prevalence. Before moving to the XIP, all Kaiabi reported with lobomycosis lived in the Arinos River or Teles Pires River regions. No new cases have occurred in Kaiabi who have only lived in XIP. The environment of the XIP is similar to Arinos River or Teles Pires River regions, but the regions have distinct watersheds.

Lobomycosis also occurs in some species of estuarine, inshore cetaceans (*Tursiops truncates*) and offshore cetaceans (*Sotalia guianensis*) (*19**–**21*). Clinical and phenotypic features of the uncultivated agent of the disease in dolphins suggested that this pathogen was the same organism, *L. loboi*. However, molecular data suggest that the cause of cutaneous lesions in dolphins is a novel strain of *P. brasiliensis*, *P. brasiliensis* var. *ceti* (*22*,*23*). Moreover, a lobomycosis-like disease among bottlenose dolphins has been reported (*24*). Bermudez et al. (*25*) hypothesized that the marine environment could be a source of the fungus in a human case they reported. Some cetaceous species inhabit the rivers of the Amazon region, including *Sotalia fluviatilis* dolphins in the Orino River in Venezuela and *Inia geoffrensis* dolphins, known as boto, in the Amazonas River in Brazil. However, these animals do not inhabit the XIP rivers, and lobomycosis has never been identified in these animals (*19*). Curiously, 1 case in Africa and 1 in Greece were reported in persons who had never traveled to South or Central America (*15*,*26*). These cases might support water as the environmental source of this fungus, but evidence of dolphin–human transmission has been weak.

Of the 63 cases of lobomycosis among the Kaiabi, most patients were male, but female Kaiabi also had a high prevalence of the disease. Early reports indicated that 32% of lobomycosis case-patients among the Kaiabi were female. Among nonindigenous women and girls, the disease occurrence ranged from 10%–12% (*27*). In addition, the increased prevalence in female Kaiabi might be because they work in close contact with the environment during activities such as small-scale farming. 

Medical reports have shown that the age of onset of the disease is typically 20–40 years of age. However, most Kaiabi case-patients began to exhibit the disease before 21 years of age. The earliest onset we noted was at 1 year of age. The susceptibility and early onset of lobomycosis among Kaiabi children can be explained by their precocious contact with wood and soil. 

Some researchers have proposed different clinical classifications for lobomycosis. For their analysis, Baruzzi et al. (*11*) adopted a modified classification, involving a localized form and multicentric form. The multicentric form is unusual and infrequent among nonindigenous populations. Other authors reported 41 cases with multicentric forms among 249 cases in nonindigenous populations (*27*). However, among the Kaiabi, the multicentric form was more common than the localized form. In analyzing the classification for sex, we noted that manifestations of the disease are quite distinct in male and female Kaiabi (Table 2). The multicentric form was more frequently seen in male case-patients and the localized form was more frequent in female case-patients.

Cellular immunodeficiency is possible in cases of lobomycosis (*28*). Different host immune responses against the fungus or relatively high exposure to the fungal agent are possible explanations for the high incidence of lobomycosis among female case-patients. However, hormonal factors could be responsible for the protection against disease dissemination in females. 

When reviewing anatomic locations of lesions, we noted that nonindigenous people more frequently have lobomycosis lesions on the outer ear. However, among the Kaiabi population, only 2 case-patients had lesions on the outer ear and many more lesions were noted on the limbs and truck. A possible reason is a variation in behavior. Nonindigenous groups tend to carry heavy burdens on their shoulders, but the Kaiabi tend to carry wood and other materials on their heads and backs. In addition, the Kaiabi do not wear shirts, so the skin of the upper body is exposed and could be exposed to the causative agent in materials they carry. The prepuce lesion was a rare localization found in 1 case.

We based our analysis on data collected by physicians who visited the XIP for >45 years. Among the Kaiabi, most of the patients with localized lobomycosis did not develop new lesions over time, but 44.1% of patients with the multicentric form had new lesions over time. Patients reported that recurrent ulcerations impaired their quality of life, and patients with the multicentric form had periods of ulcerations more frequently than those with the localized form. We did not observe any change in ulcer healing or worsening related to weather or seasons of the year. Among all lobomycosis cases worldwide, 4 reports of progression to squamous cell carcinoma have occurred; 2 were in the Kaiabi population, and both died from metastasis (*8*). Outside of the Kaiabi population, no deaths from complications of lobomycosis have been reported. In addition, among indigenous populations living in the XIP, lobomycosis greatly affects quality of life; those who have it are stigmatized, and the disease has been known as Kaiabi leprosy in the past*.*

Treatment is a challenge, and systemic drugs are not effective (*29*). However, many treatment attempts have been reported in some case-patients. The medical literature reports some isolated positive results with itraconazole, clofazimine, leprosy multidrug therapy, or posaconazole (*27*,*30*). Other approaches, such as surgical excision, cryosurgery, and electrosurgery, should be considered in addition to drug treatment. However, no known effective treatment for lobomycosis, especially the multicentric form, has been found (*30*). Among the Kaiabi, treatment by surgery was successful in some localized cases without recurrence in a 30-year follow-up. 

In conclusion, lobomycosis is a rare disease that affects certain geographic regions, especially in countries with low socioeconomic status (*31*), but its prevalence among the Kaiabi population in Brazil is exceptionally high. The 63 cases we identified among this population represent 11.5% of all cases of lobomycosis known in the world since the disease was identified. Our clinical and epidemiologic analysis shows cases among the Kaiabi have particular characteristics, including a high incidence of female case-patients, early onset in persons <21 years of age, a high incidence of the multicentric form in male case-patients, and a high incidence of the localized form in female case-patients. Little is known about the prevalence of lobomycosis and the effective treatment remains a challenge, especially for the multicentric form. Phylogenetic analysis of *L. loboi* could help in the development of treatments for this fungal infection.

## References

[1] Arenas CM, Rodriguez-Toro G, Ortiz-Florez A, Serrato I. Lobomycosis in Soldiers, Colombia. Emerg Infect Dis. 2019;25:654–60. 10.3201/eid2504.181403 30882301 PMC6433027

[2] Queiroz-Telles F, Fahal AH, Falci DR, Caceres DH, Chiller T, Pasqualotto AC. Neglected endemic mycoses. Lancet Infect Dis. 2017;17:e367–77. 10.1016/S1473-3099(17)30306-7 28774696

[3] Beltrame A, Danesi P, Farina C, Orza P, Perandin F, Zanardello C, et al. Case report: molecular confirmation of lobomycosis in an Italian traveler acquired in the Amazon region of Venezuela. Am J Trop Med Hyg. 2017;97:1757–60. 10.4269/ajtmh.17-0446 29016315 PMC5805066

[4] Elsayed S, Kuhn SM, Barber D, Church DL, Adams S, Kasper R. Human case of lobomycosis. Emerg Infect Dis. 2004;10:715–8. 10.3201/eid1004.030416 15200867 PMC3323076

[5] Lobo J. A case of blastomycosis, produced by a new species, found in Recife [in Portuguese]. Rev Med Pernamb. 1931;1:763–75.

[6] Carvalho KA, Floriano MC, Enokihara MM, Mascarenhas MR. Jorge Lobo’s disease. An Bras Dermatol. 2015;90:586–8. 10.1590/abd1806-4841.20153603 26375233 PMC4560553

[7] Camargo ZP, Baruzzi RG, Maeda SM, Floriano MC. Antigenic relationship between *Loboa loboi* and *Paracoccidioides brasiliensis* as shown by serological methods. Med Mycol. 1998;36:413–7. 10.1080/02681219880000651 10206752

[8] Baruzzi RG, Rodrigues DA, Michalany NS, Salomão R. Squamous-cell carcinoma and lobomycosis (Jorge Lobo’s disease). Int J Dermatol. 1989;28:183–5. 10.1111/j.1365-4362.1989.tb02459.x 2707943

[9] Lobomycosis R-TG. Int J Dermatol. 1993;32:325–32. 10.1111/j.1365-4362.1993.tb01466.x 8505156

[10] Florian MC, Tomimori J, de Mendonça SBM, Rodrigues DA. Dermatological atlas of indigenous people. Cham (Switzerland): Springer; 2017.

[11] Baruzzi RG, Castro RM, D’Andretta C Jr, Carvalhal S, Ramos OL, Pontes PL. Occurrence of Lobo’s blastomycosis among “Caiabi,” Brazilian Indians. Int J Dermatol. 1973;12:95–9. 10.1111/j.1365-4362.1973.tb00011.x 4697345

[12] Lacaz CS, Baruzzi RG, Rosa MCB. Jorge Lobo’s disease [in Portuguese]. São Paulo (Brazil): Editora da Universidade de São Paulo; 1986.

[13] Baruzzi RG, Marcopito LF, Vicente LS, Michalany NS. Jorge Lobo’s disease (keloidal blastomycosis) and *tinea imbricata* in Indians from the Xingu National Park, Central Brazil. Trop Doct. 1982;12:13–5. 10.1177/004947558201200106 7071925

[14] Baruzzi RG, Marcopito LF, Michalany NS, Livianu J, Pinto NRS. Early diagnosis and prompt treatment by surgery in Jorge Lobo’s disease (keloidal blastomycosis). Mycopathologia. 1981;74:51–4. 10.1007/BF00441441 7242649

[15] Papadavid E, Dalamaga M, Kapniari I, Pantelidaki E, Papageorgiou S, Pappa V, et al. Lobomycosis: A case from Southeastern Europe and review of the literature. J Dermatol Case Rep. 2012;6:65–9. 10.3315/jdcr.2012.1104 23091581 PMC3470791

[16] Pereira Filho MJ. The fungi of the disease of Adolfo Lutz, of the disease of Jorge Lobo, and that of the blastomycosis of the Indians of Alto Xingu (Central Brazil State of Mato Grosso). [in Portuguese]. Rev Med Rio Gd Sul. 1957;14:10–64.

[17] Nery-Guimarães F. Inoculations in hamsters of South American blastomycosis (Lutz disease), queloidian blastomycosis (Lôbo disease) and blastomycosis of Tapajós-Xingu Indians [in Portuguese]. Hospital (Rio J). 1964;66:581–93. 14202717

[18] Lacas CS. Jorge Lobo (1900-1979). J Cutan Pathol. 1981;8:75–6. 10.1111/j.1600-0560.1981.tb00987.x 7009664

[19] Francesconi VA, Klein AP, Santos AP, Ramasawmy R, Francesconi F. Lobomycosis: epidemiology, clinical presentation, and management options. Ther Clin Risk Manag. 2014;10:851–60. 10.2147/TCRM.S46251 25328400 PMC4199563

[20] Rotstein DS, Burdett LG, McLellan W, Schwacke L, Rowles T, Terio KA, et al. Lobomycosis in offshore bottlenose dolphins (*Tursiops truncatus*), North Carolina. Emerg Infect Dis. 2009;15:588–90. 10.3201/eid1504.081358 19331739 PMC2671444

[21] Paniz-Mondolfi AE, Sander-Hoffmann L. Lobomycosis in inshore and estuarine dolphins. Emerg Infect Dis. 2009;15:672–3. 10.3201/eid1504.080955 19331770 PMC2671448

[22] Vilela R, Bossart GD, St Leger JA, Dalton LM, Reif JS, Schaefer AM, et al. Cutaneous granulomas in dolphins caused by novel uncultivated *Paracoccidioides brasiliensis.* Emerg Infect Dis. 2016;22:2063–9. 10.3201/eid2212.160860 27869614 PMC5189160

[23] Seyedmousavi S, de Hoog GS, Guillot J, Verweij PE. Emerging and epizootic fungal infections in animals. Basel (Switzerland): Springer; 2018.

[24] Van Bressem MF, Simões-Lopes PC, Félix F, Kiszka JJ, Daura-Jorge FG, Avila IC, et al. Epidemiology of lobomycosis-like disease in bottlenose dolphins *Tursiops* spp. from South America and southern Africa. Dis Aquat Organ. 2015;117:59–75. 10.3354/dao02932 26575156

[25] Bermudez L, Van Bressem MF, Reyes-Jaimes O, Sayegh AJ, Paniz-Mondolfi AE. Lobomycosis in man and lobomycosis-like disease in bottlenose dolphin, Venezuela. Emerg Infect Dis. 2009;15:1301–3. 10.3201/eid1508.090347 19751598 PMC2815985

[26] Al-Daraji WI, Husain E, Robson A. Lobomycosis in African patients. Br J Dermatol. 2008;159:234–6. 10.1111/j.1365-2133.2008.08586.x 18460023

[27] Woods WJ, Belone AF, Carneiro LB, Rosa PS. Ten years experience with Jorge Lobo’s disease in the state of Acre, Amazon region, Brazil. Rev Inst Med Trop São Paulo. 2010;52:273–8. 10.1590/S0036-46652010000500010 21049233

[28] Xavier MB, Ferreira MM, Quaresma JA, de Brito A. HIV and lacaziosis, Brazil. Emerg Infect Dis. 2006;12:526–7. 10.3201/eid1203.051426 16710984 PMC3291465

[29] Araújo MG, Cirilo NS, Santos SNMBD, Aguilar CR, Guedes ACM. Lobomycosis: a therapeutic challenge. An Bras Dermatol. 2018;93:279–81. 10.1590/abd1806-4841.20187044 29723380 PMC5916408

[30] Bustamante B, Seas C, Salomon M, Bravo F. Lobomycosis successfully treated with posaconazole. Am J Trop Med Hyg. 2013;88:1207–8. 10.4269/ajtmh.12-0428 23546805 PMC3752824

[31] de Souza MN, Schlosser AR, da Silva-Nunes M. Lobomycosis of the lower limb in an Amazonian patient. Am J Trop Med Hyg. 2015;93:675–6. 10.4269/ajtmh.14-0748 26446617 PMC4596579

